# Nutritional Therapies and Their Influence on the Intestinal Microbiome in Pediatric Inflammatory Bowel Disease

**DOI:** 10.3390/nu14010004

**Published:** 2021-12-21

**Authors:** Lara Hart, Charlotte M. Verburgt, Eytan Wine, Mary Zachos, Alisha Poppen, Mallory Chavannes, Johan Van Limbergen, Nikhil Pai

**Affiliations:** 1 Department of Paediatrics, Division of Paediatric Gastroenterology & Nutrition, McMaster University, Hamilton, ON L8N 3Z5, Canada; lara.hart@medportal.ca (L.H.); zachosm@mcmaster.ca (M.Z.); 2 McMaster Children’s Hospital, Hamilton, ON L8N 3Z5, Canada; 3 Department of Pediatric Gastroenterology and Nutrition, Amsterdam University Medical Centers, Emma Children’s Hospital, 1105 AZ Amsterdam, The Netherlands; c.m.verburgt@amsterdamumc.nl (C.M.V.); j.e.vanlimbergen@amsterdamumc.nl (J.V.L.); 4 Tytgat Institute for Liver and Intestinal Research, Amsterdam Gastroenterology Endocrinology and Metabolism, Amsterdam University Medical Centers, University of Amsterdam, 1105 AZ Amsterdam, The Netherlands; 5 Amsterdam Reproduction & Development Research Institute, Amsterdam University Medical Centers, Emma Children’s Hospital, 1105 AZ Amsterdam, The Netherlands; 6 Edmonton Paediatric IBD Clinic, Division of Paediatric Gastroenterology and Nutrition, Departments of Paediatrics & Physiology, University of Alberta, Edmonton, AB T6G 2R3, Canada; wine@ualberta.ca; 7 College of Medicine and Health, University College Cork, T12 K8AF Cork, Ireland; alisha.poppen@gmail.com; 8 Department of Paediatrics, Division of Paediatric Gastroenterology and Nutrition, Children’s Hospital of Los Angeles, Los Angeles, CA 90027, USA; mchavannes@chla.usc.edu; 9 Department of Paediatrics, Dalhousie University, Halifax, NS B3H 4R2, Canada; 10 Farncombe Family Digestive Health Research Institute, McMaster University, Hamilton, ON L8N 3Z5, Canada; 11 Centre for Metabolism, Obesity and Diabetes Research, McMaster University, Hamilton, ON L8N 3Z5, Canada

**Keywords:** microbiome, nutrition, diet, EEN, PEN, SCD, CDED, CD-TREAT, Mediterranean, UCED, FODMAP, IBD, UC, CD

## Abstract

Inflammatory bowel disease (IBD) is a chronic, autoimmune disorder of the gastrointestinal tract with numerous genetic and environmental risk factors. Patients with Crohn’s disease (CD) or ulcerative colitis (UC) often demonstrate marked disruptions of their gut microbiome. The intestinal microbiota is strongly influenced by diet. The association between the increasing incidence of IBD worldwide and increased consumption of a westernized diet suggests host nutrition may influence the progression or treatment of IBD via the microbiome. Several nutritional therapies have been studied for the treatment of CD and UC. While their mechanisms of action are only partially understood, existing studies do suggest that diet-driven changes in microbial composition and function underlie the diverse mechanisms of nutritional therapy. Despite existing therapies for IBD focusing heavily on immune suppression, nutrition is an important treatment option due to its superior safety profile, potentially low cost, and benefits for growth and development. These benefits are increasingly important to patients. In this review, we will describe the clinical efficacy of the different nutritional therapies that have been described for the treatment of CD and UC. We will also describe the effects of each nutritional therapy on the gut microbiome and summarize the strength of the literature with recommendations for the practicing clinician.

## 1. Introduction

Immune dysfunction drives the pathogenesis of inflammatory bowel disease (IBD), including ulcerative colitis (UC) and Crohn’s disease (CD). Current therapies to treat IBD include long-term medications such as immunosuppressive therapies and monoclonal antibodies, which are typically studied in short-term induction of remission studies. Sustained remission, beyond one year, is more difficult to achieve and requires careful assessment of benefits and risks for patients [1,2].

The role of the microbiome in IBD has also been increasingly recognized. Animal models have described how dysbiosis can lead to the development of IBD in a genetically susceptible host, and similar associations have been found in human studies [3]. Germ-free environments have been demonstrated to prevent colitis in genetically susceptible mice [4]. Transfer of proinflammatory bacteria from diseased mice to healthy mice induces inflammation [5,6]. Disease activity is most commonly seen with high concentrations of bacteria (distal small bowel and colon) or relative stasis of fecal material, and fecal diversion is an effective strategy in the management of CD, with remission occurring in the excluded segment of the bowel [7,8]. Bidirectional microbial interactions via the gut–brain axis continue to be described, including effects of intestinal inflammation on the permeability of the blood–brain barrier and potential compensatory constriction of the choroid plexus vascular barrier [9,10]. The development of microbiota-based therapeutics is still in its relative infancy, but emerging treatments such as fecal microbiota transplant (FMT) from healthy donors to affected recipients for the treatment of UC have shown efficacy in both adults and children [11,12].

Multiple studies have also demonstrated that diet has a profound impact on the intestinal microbiome. An acute change in diet, such as from strictly animal-based to plant-based nutrition, has been shown to alter microbial composition within just 24 h, with reversion to baseline within 48 h of diet discontinuation [13]. Intestinal dysbiosis has been seen in studies of malnourished children from Bangladesh and Malawi [14,15]. Transferring this intestinal microbiota from malnourished children into germ-free mice recapitulated the malnourished phenotype to the mice causing impaired growth [15]. Carefully selecting a microbiota-directed therapeutic food strategy has been shown to correct dysbiosis, correct malnutrition and restore growth [16,17].

An intricate relationship exists between diet, the intestinal microbiome, and immune homeostasis, especially in IBD. Many patients desire therapeutic diets as an alternative or adjunct to their medical therapies, as current treatment approaches still mainly focus on suppressing the immune system. Indeed, the role of diet in the management of IBD is one of the most common questions that patients ask their providers.

### Basic Concepts of Nutrition in Intestinal Inflammation

The rising incidence of IBD has been linked to clustered effects of higher socioeconomic development, including greater consumption of a Western Diet (WD) [18,19,20]. Numerous chronic disease conditions have been associated with the WD, including obesity, hypertension, insulin resistance, and chronic kidney disease [21,22,23]. Dietary intake is associated with enrichment and reduction of multiple proinflammatory and anti-inflammatory taxa of the gut microbiome [24]. In turn, WD intake has been associated with an increased risk of colitis and flare occurrence in IBD [20,25]. Multiple studies describe how IBD patients often tend to avoid certain foods during active disease flares and periods of remission, even independent of physician guidance [26,27].

The typical WD is characterized by higher amounts of processed food, red meat, fat, sugar, emulsifier, and additive exposure, as well as decreased amounts of fibre, fruit, and vegetables [28]. Low fibre, high sugar, food additives and other typical components of the WD have all been described to increase intestinal permeability, potentially leading to increased susceptibility to colitis [29,30,31,32]. Fibre is a vital substrate for the production of short-chain fatty acids (SCFA) through microbial fermentation, which plays a vital role in modulating the immune response and serves as a primary energy substrate for intestinal epithelial cells [29]. Higher amounts of animal protein and red meat intake have been shown to alter the microbial composition and have been linked to increased incidence of IBD and worsening of colitis severity in animal models [13,33,34]. Similar trends are also seen with emulsifiers, which can promote intestinal inflammation through bacterial translocation and food additives; this can impair antibacterial responses and suppress antimicrobial defence mechanisms [32,35].

**Figure 1:**
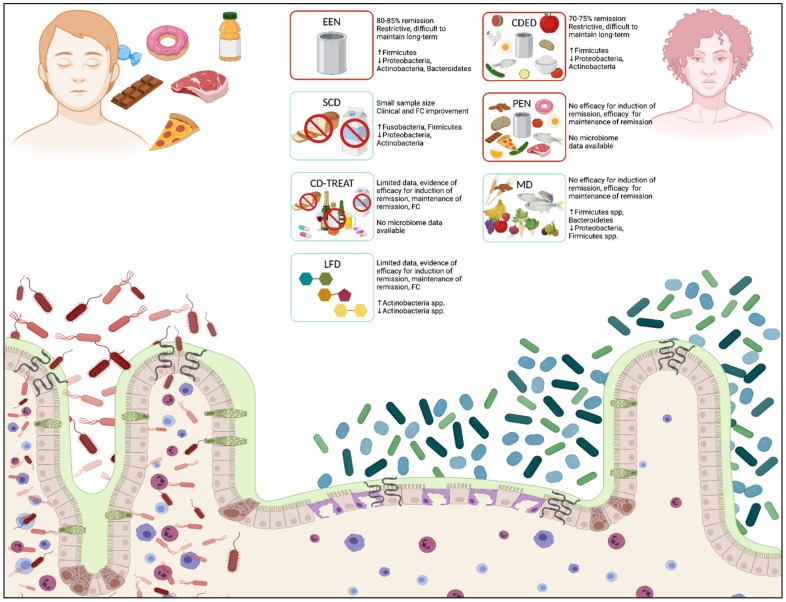
Effects of diet and nutritional therapies on intestinal microbiota and mucosal immune response. Dietary influences affect the intestinal microbiota and impact mucosal immune responses through numerous direct and indirect mechanisms. A typical western diet (LEFT), consisting of high amounts of processed foods, red meat, high-sugar foods, and prepackaged foods, can induce a relative dysbiosis in the intestinal lumen, increase intestinal permeability at epithelial junctions, and promote direct bacterial translocation across the epithelial membrane. Nutritional therapies in patients with IBD (RIGHT) support a healthier composition of bacterial taxa, which decreases inflammation at the intes-tinal epithelium, reduces intestinal permeability, and decreases mucosal inflammation. Multiple nutritional therapies are proposed for the treatment of CD and UC, including EEN, CDED, and PEN (diets with proof of clinical efficacy in IBD, outlined in red), and SCD, CD-TREAT, MD, and LFD (diets with no proof of clinical efficacy in IBD, outlined in blue). Diets depict major food groups that are permitted (foods/carbohydrate structures presented within each box) and re-stricted (foods crossed out within each box). Clinical efficacy and significant changes to bacterial taxa (phylum level) are described for each diet. Crohn’s disease (CD); Crohn’s disease exclusion diet (CDED); Crohn’s disease treatment-with-eating diet (CD-TREAT); exclusive enteral nutrition (EEN); fecal calprotectin (FC); inflammatory bowel disease (IBD); low fermentable oligo-, di-, monosaccharides and polyol (FODMAP) diet (LFD); Mediterranean diet (MD); partial enteral nutrition (PEN); specific carbohydrate diet (SCD); ulcerative colitis (UC). (Created in BioRender.com, accessed on 1 December 2021) [36].

Given the effects of dietary influences on intestinal permeability, host immunity, microbial composition, and the development of colitis, different dietary therapies have been developed (see: Figure 1). These diets involve complete or partial exclusion of specific dietary components to induce or maintain remission in paediatric IBD. In this review, we will explore the role of several nutritional therapies in the treatment of UC and CD, summarize their efficacy, and describe their impacts on the intestinal microbiome. This will start with exclusive enteral nutrition (EEN) and include recently introduced dietary therapies.

## 2. Diets with Proof of Clinical Efficacy for IBD

### 2.1. Exclusive Enteral Nutrition (EEN)

Despite the fact that diet is being proposed as a key factor in the pathogenesis of IBD, very few diets have demonstrated clear therapeutic benefits. The best known and most evidence-supported dietary therapy in IBD is EEN, which is currently considered the first-line therapy for induction of remission in paediatric luminal CD (nonstricturing, nonpenetrating), following the European Crohn’s and Colitis Organization (ECCO) and European Society for Paediatric Gastroenterology Hepatology and Nutrition (ESPGHAN) guidelines [37]. EEN involves a complete and balanced liquid meal replacement and the exclusion of all foods and beverages, usually for a period of six to eight weeks [38,39]. Commonly used EEN formulas include Ensure/Ensure Plus^®^, Modulen IBD^®^, Pediasure^®^, or Nutren/Nutren Junior^®^. Both elemental and polymeric formulae have been shown to be effective and can be provided orally or by nasogastric tube feeding if not orally tolerated [40,41]. EEN is also effective for active CD regardless of disease location (including upper GI tract, small bowel, and colon), disease severity, and new diagnosis versus relapse or flare [38,39,40,41,42].

#### 2.1.1. Mechanisms of Action of EEN

The exact mechanisms of action of EEN remain unknown and involve several synergistic factors. These include the exclusion of many “offending foods” or other potentially damaging dietary components (such as food preservatives and emulsifiers), direct effects on host immune response, microbial modulation, and enhanced intestinal barrier function with decreased inflammation; these mechanisms have been reviewed extensively in other papers [28,39,43]. Direct benefits from the availability of a rich and balanced diet (as enterocytes obtain most of their nutrients from the luminal content) have also been suggested to contribute to the effectiveness of EEN, with specific effects on enterocyte differentiation [44].

The principles of dietary exclusion are common to other diets reviewed below (i.e., Mediterranean diet, specific carbohydrate diet, and CD-TREAT diet) and are best supported by the lower efficacy of partial enteral nutrition (PEN), where only 15% of children achieved remission with 50% formula and 50% regular diet [45,46,47]. Some dietary components have already received attention as potentially “‘offending foods”; however, most studies supporting these effects are conducted in animal models or cells in a culture, given the difficulty of conducting dietary research in humans with IBD [48].

One example is emulsifiers, which have been shown to have negative impacts on the gut environment, including impaired mucosal immunity, intestinal permeability, and the microbiome [49]. A 2015 study by Chassaing et al. in mice showed the ability of two commonly used emulsifiers, carboxymethylcellulose and polysorbate-80, to induce inflammation and metabolic changes, mediated by gut microbes [32]. Multiple studies have investigated the negative effects of a “WD,” including animal-based proteins and fats, and have shown that replacing these with plant-based food sources is beneficial in IBD animal models. Again, these have been hypothesized to be mediated by effects on the microbiota [37].

The theory that processed food is a primary driver of inflammation in IBD has been somewhat challenged by work published by Logan et al. in 2020, who demonstrated that some components of successful EEN formulae include preservatives and processed compounds [50]. Furthermore, the role of fibre (lack of fibre) in IBD is supported by multiple observations, such as damage to the intestinal mucosal layer when gut microbes are starved of fibres [29]. Following these observations, one would expect that a liquid formula diet (as used in EEN) that is low in fibre would not confer benefit; however, dietary fibres are diverse and have various effects on bowel homeostasis and IBD pathogenesis [51]. These examples highlight the complexity of nutritional science in IBD and the need for better-controlled studies.

#### 2.1.2. Clinical Efficacy of EEN

Data supporting the effectiveness of EEN have been reviewed in several meta-analyses and indicate at least equivalent clinical response to steroids (18 studies, *N* = 1329, OR = 1.35, 95% CI: 0.90–2.10, favouring EEN; *p* = 0.14) with most studies showing a remarkable 80–85% remission rate (see: Table 1) [52,53]. EEN was superior to steroids for inducing mucosal healing in a subset of the studies (OR = 4.5, 95% CI: 1.64–12.32) [52,53]. Emerging data from studies across multiple centres, including adult patients with CD, have shown similar results [54,55,56]. Positive effects of EEN (and other dietary therapies) can be seen as early as two weeks after initiation, including improvement in clinical disease activity scores, fecal calprotectin (FC), C-reactive protein (CRP), and extraintestinal manifestations of CD such as bone health [57,58,59]. EEN is recommended as first-line therapy to induce remission in paediatric CD [42]. While these recommendations differ in adult CD management [2,60], a prospective, open-label trial from 2018 in Japan assessing the role of combination infliximab dose escalation with elemental diet versus infliximab dose escalation alone had to be stopped early due to clear benefits in the combination therapy over monotherapy treatment group in the midterm analysis [61].

Early experience suggested that EEN was more likely to be effective in patients with small bowel disease than colonic CD, based on higher stooling frequency during initial treatment in colonic CD, likely owing to osmotic effects of formula feeds in the affected colon. The effectiveness of EEN has since been demonstrated regardless of the site [62]. Nevertheless, the experience of EEN for the treatment of UC is limited. A recent open-label RCT from 2021 by Sahu et al. assessed the role of EEN in acute severe ulcerative colitis (ASUC) [63]. A total of 62 adult patients were randomized to semielemental formula for seven days along with intravenous (IV) corticosteroids or IV corticosteroids alone. Patients receiving EEN + IV corticosteroids compared to IV corticosteroids alone had lower rates of treatment failure (19% versus 43%; *p* = 0.04), shorter length of stay in hospital (median 10 versus 13 days; *p* = 0.04), and a lower composite outcome of colectomy/hospitalization at six months (16% versus 39%; *p* = 0.045).

**Table 1:** Reported clinical outcomes by nutritional therapy.

Type of Diet	Study Design	Main Outcomes	Limitations OF Studies	Limitations of Dietary Therapy
EEN	Multiple study designs, including two systematic reviews [52,64].	80–85% remission rate Equivalent to CS for clinical remission Small studies show EEN is superior to CS for mucosal healing [52,65].	Adult studies have shown decreased efficacy in ITT analyses due to high patient dropout rates	Very restrictive Difficult to maintain long term due to issues of tolerability Poor palatability
PEN [66,67,68]	Multiple study designs, induction of remission One systematic review, maintenance of remission	No evidence of efficacy for induction of remission The systematic review demonstrates efficacy for maintenance of remission	No consistent definition of PEN (percent caloric intake) Variable methodologies used across studies	Can be restrictive Difficult to maintain long term
CDED [48,69,70]	RCT: EEN versus CDED Two prospective, open-label studies	Week Six: EEN and CDED are both effective at achieving clinical remission Week 12: significantly higher CS-free remission in CDED group (76%) Earlier studies: 70–75% remission rate in children at week six	The primary outcome of RCT was tolerability Small numbers, nonrandomized, inconsistent protocol (not all participants took PEN) in prospective, open-label studies	Most effective for mild-moderate luminal CD The induction phase of treatment is relatively restrictive
SCD [68]	Exploratory multi-omic pilot study: SCD versus mSCD versus whole food	All patients showed clinical improvement and FC improvement SCD showed the greatest clinical improvement mSCD showed the greatest FC change Cannot conclude significance with sample size	Small sample size: 18 patients recruited; 10 patients completed the study Baseline mild disease (normal CRP, normal/mild increase ESR) Significant side effects among recipients of WF diet	Very restrictive May be difficult to adhere to without support for meal preparation
CD-TREAT [46]	RCT: EEN versus CD-TREAT in healthy adults Five children received CD-TREAT	80% (four out of five) clinical improvement 60% (three out of five) clinical remission, improved FC	Very small sample size	Easier diet to follow Limited data
MD [47]	RCT: MD versus SCD	No significant difference in clinical symptoms, FC values between MD and SCD	The sample included patients with primarily mild disease Not all patients had elevated FC at baseline Lack of control arm	None Easiest diet to follow Miscellaneous health benefits may occur for conditions other than IBD
LFD [71,72]	Two RCTs	52% decrease in symptoms 34% decrease in FC No effect on CRP	Small sample size Patient-reported outcomes (subjectivity, potential placebo effect) Patients primarily in remission (or with mild disease)	Can be restrictive

Crohn’s disease (CD); Crohn’s disease exclusion diet (CDED); Crohn’s disease treatment-with-eating diet (CD-TREAT); C-reactive protein (CRP); corticosteroids (CS); exclusive enteral nutrition (EEN); fecal calprotectin (FC); low fermentable oligo-, di-, monosaccharides and polyol (FODMAP) diet (LFD); inflammatory bowel disease (IBD); intention-to-treat (ITT); Mediterranean diet (MD); partial enteral nutrition (PEN); randomized controlled trial (RCT); specific carbohydrate diet (SCD); modified specific carbohydrate diet (mSCD); whole-food (WF).

One of the main advantages of EEN is its superior safety profile (particularly compared to steroids) and the added benefit of improving growth and nutrition [73]. However, EEN is difficult to tolerate and poses considerable challenges to patients and families, primarily due to poor palatability, the monotony of the diet, and the cost [41,74]. EEN requires considerable resources and is most effective when supported by a multidisciplinary IBD care team [58,61,63].

#### 2.1.3. Effects of EEN on the Microbiome

Many of these studies help focus attention on the role of gut microbes in the pathogenesis of IBD, especially the presence of pathobionts, and how EEN might dampen such adverse effects and reduce pathobionts. Observed changes in microbiota with EEN are diverse and depend on study design and technical consideration (see: Table 2). Nonetheless, some taxonomic alterations are reproducible [38,75]. These changes can be rapid, occurring even within one week of initiating EEN therapy [76]. Indeed, dramatic effects are observed, some of which are counterintuitive to our understanding of the impact of microbiota on CD pathogenesis. For example, EEN appears to reduce microbial diversity, lower SCFA concentrations (including butyrate), and reduce *Faecalibacterium prausnitzii*, which is usually considered beneficial in IBD [77]. In another study, responders to EEN showed lower bacterial richness than nonresponders [78]. Quince et al. showed a decrease in Shannon diversity with EEN, but this returned to pretreatment levels two months after EEN was stopped, as did decreases in *Bifidobacterium, Ruminococcus, and Faecalibacterium* [79]. It is hypothesized that some of these decreases in specific taxa and diversity are simply due to the lack of fibre in EEN [80,81]. Furthermore, a baseline reduction in dysbiosis-associated taxa could potentially be a marker of better response to EEN, where patients who respond to therapy may subsequently have paradoxical increases in pathobiont taxa.

Several studies have attempted to correlate response to EEN with changes in microbes. How the baseline microbes respond to such a drastic diet change could determine the likelihood of therapy response [84]. Patients who demonstrated decreases in *Bacteroides* and *Prevotella* species with EEN treatment improved clinically, and these changes in microbiota persisted for several months after completing EEN [81]. While bacterial composition has been shown to be predictive in several studies, it is critical to differentiate it from microbial function (measured using metagenomics), which highlights potential mechanisms of action [85]. A recent study using stool metabolomics showed that amino acids, primary bile salts, trimethylamine, and cadaverine are reduced with EEN in children with Crohn’s disease, likely mediated by microbes, and it is believed that this reduction best predicts response to EEN [86,87].

It remains unclear whether microbial shifts observed with EEN are responsible for the effectiveness of therapy or are a consequence of successful therapy and resolution of inflammation. One recent study showed an improvement in microbial diversity with both EEN and steroids, associated with response to therapy, suggesting that at least some of the microbial changes may simply be due to reduced inflammation [86]. Collectively, these studies do suggest that EEN does act through compositional and functional microbial changes, but much remains to be understood in this growing field of research.

#### 2.1.4. Summary

EEN has been shown in numerous high-quality studies to be an effective treatment for the induction of remission in CD. Large systematic reviews have indicated that EEN and CS are equally effective in inducing remission in CD, and EEN is recommended as first-line therapy for induction for paediatric CD. A recent open-label RCT suggests a short course of EEN and CS in combination may be more effective than CS alone for ASUC. While there are small studies to indicate EEN is superior to CS for mucosal healing, further work is needed before this can be concluded.

### 2.2. Partial Enteral Nutrition (PEN)

In contrast to EEN, partial enteral nutrition (PEN) uses a nutritionally balanced liquid formula to provide less than 80% of daily caloric requirements. PEN is complemented by a supplemental diet of solid food. PEN can be administered with or without a directed exclusion diet [28,69].

#### 2.2.1. Mechanisms of Action of PEN

The mechanism of action of PEN remains unknown, but similar mechanisms underlying the effectiveness of EEN mediate the effects of PEN. Preclinical studies assessing modulation of colonocyte inflammation using polymeric formula demonstrate improved cell viability and survival with exposure to polymeric formula, compared with cells stimulated by tumour necrosis factor-alpha (TNF-α) [88]. Additionally, the same group demonstrated both enriched nuclear factor kappa B subunit 1 (NFκB1)-associated pathways via microarray analysis after applying the polymeric formula in TNF-α stimulated colonocytes and significant downregulation of TNF signalling. In a small study of 34 patients, Marques et al. described metabolomic differences between paediatric patients with minimally active or quiescent disease receiving PEN for maintenance of remission as compared to those receiving a regular diet [89]. Baseline differences in metabolomic profiles between treatment groups may have affected results. Further research is needed in this area, including a head-to-head comparison of microbial composition and function in patients receiving PEN versus a regular diet.

#### 2.2.2. Clinical Efficacy of PEN

There is considerable heterogeneity among studies assessing the clinical effectiveness and efficacy of PEN. This may stem from the lack of standardization with the amount administered (percentage of calories per day), type of food allowed, and duration of treatment across centers [68]. Without a concomitant exclusion diet, there is a lack of convincing data supporting the use of PEN to induce clinical remission in CD.

Despite this, PEN has been used successfully to maintain clinical remission in CD [45,66,67,90,91,92,93,94,95,96]. Additionally, patients on PEN show improvements in nutritional status, defined by improved weight gain, linear growth and levels of micronutrients such as vitamins A, B12, D and E and minerals [92,93,94,96,97]. A systematic review (*n* = 429) of prospective studies assessing the effectiveness of PEN for maintenance of remission in CD demonstrated that PEN was superior at preventing clinical relapse (RR 0.67, 95% CI: 0.54–0.82, *p* < 0.01) and achieving clinical remission (RR 1.32, 95% CI: 1.07–1.64; *p* = 0.01) than those not receiving nutritional therapy [68].

A recent abstract presented at the 2021 European Crohn’s and Colitis Organization’s annual congress reported 100 treatment-naïve paediatric CD patients randomized to cyclic EEN (N = 49), or daily supplementary nutrition (PEN) combined with normal access to food (N = 51) for 12 consecutive months [98]. Fifty-one percent (25/49) of patients in the cyclic EEN group versus 24% (12/51) in the PEN group (*p* = 0.004) remained in remission at 12 months, suggesting that paediatric patients with CD can be maintained in remission with cyclic EEN without immunosuppression or biologics. Patients in the PEN group showed a relapse rate of 76%, suggesting PEN was not as effective as cycling EEN, nor was an effective long-term maintenance strategy. More extensive multicentre randomized–controlled trials (RCT) are needed to compare PEN protocols and define minimal percent of total calories required from enteral formula for clinical benefit in paediatric patients (see: Table 3).

#### 2.2.3. Summary

Current data do not demonstrate the efficacy of PEN for the induction of remission in CD. However, PEN may have a role in the maintenance of remission for paediatric CD based on a recent systematic review. Existing studies have varied in methodology and inclusion criteria, particularly in defining percent calories that constitute “partial” enteral nutrition. Further research applying a common, standardized methodology will be required to further describe the role of EEN as maintenance therapy [98].

### 2.3. Crohn’s Disease Exclusion Diet (CDED)

Given the success of EEN at inducing remission in Crohn’s disease, significant work has been devoted to identifying those components of a “normal diet” that are proinflammatory and should be excluded. Levine et al. first described the Crohn’s Disease Exclusion Diet (CDED) in 2014 as a diet that allows patients to eat whole foods while concomitantly reducing exposure to inflammatory dietary factors [70].

CDED is thought to act through three main mechanisms that underpin intestinal inflammation in CD: improving intestinal permeability, decreasing adherence and reducing translocation of bacteria, and altering the intestinal microbiota to a less inflammatory composition [70]. CDED is a high protein, low-fat diet that includes foods such as chicken, fish, eggs, rice, potatoes and various fruit and vegetables. CDED restricts processed foods, animal fat, gluten and dairy, as well as certain additives such as emulsifiers, maltodextrins, carrageenan and sulphites [69,70].

The dietary intervention consists of six weeks of CDED plus PEN (50% CDED, 50% PEN). After that, the patient continues with an additional six weeks of CDED with 25% PEN, with more foods progressively introduced back into the diet [70]. This intervention has been highly effective for mild-to-moderate luminal CD, as well as for patients who have had a loss of response to anti-TNF biologic treatments [69,70,99]. For severe CD, patients receiving the CDED can receive two weeks of exclusive enteral nutrition before commencing CDED protocols [99]. A third stage (maintenance) is currently being studied and involves progressively introducing more foods every six weeks.

#### 2.3.1. Clinical Efficacy of CDED

The first results of CDED were published in 2014 with 33 children and 14 adults [70]. Participants followed a 12-week protocol, as described above. Remission was defined as Paediatric Crohn’s Disease Activity Index (PCDAI) <7.5 or Harvey Bradshaw index (HBI) ≤3. Taken together, remission was seen in 70% of children and 69% of adults at week six, 84% of patients maintained remission at week 12, and 70% normalized their CRP. Disease severity was the only predictor of lack of response. Only 33% of patients with severe disease at baseline (PCDAI > 40, HBI > 9) reached remission, compared with 71–75% of patients with mild-to-moderate patients (PCDAI 7.5–27.5 or HBI 4–6, mild disease; PCDAI 30–37.5 or HBI 7–8, moderate disease).

A second study was published in 2017 and assessed the use of CDED in patients who lost response to infliximab or adalimumab [99]. The study included 11 adults and 10 children, and all participants received 12 weeks of CDED with 50% PEN (rather than the two-step protocol described above). There was a significant decrease in HBI and CRP at weeks six and twelve and an improvement in albumin at week six. Clinical response occurred in 90% of patients at week six, and clinical remission in 62%. The authors also describe 18 patients who received CDED alone (without PEN), with 77% reaching clinical remission (14 patients).

In 2019, the first multicentre RCT was conducted exclusively in children comparing CDED + PEN to EEN for remission induction [69]. A total of 74 patients with mild to moderate CD were included and either received the 2-phase 12-week CDED protocol (as described above) or six weeks of EEN followed by 75% unrestricted diet +25% PEN, gradually returning to normal diet at week 12. The primary outcome was the tolerability of the intervention. Tolerability was significantly higher in the CDED group (97% CDED versus 74% EEN). At week six, CDED and EEN were found to be equally effective at inducing remission, while at week 12, there was a significantly higher proportion of patients in corticosteroid-free sustained remission in the CDED group (76% CDED versus 45% EEN groups); 87.5% of patients in remission at week six, maintained remission at week 12. This was associated with sustained improvement in markers of inflammation (CRP and FC) in the CDED group. Data from this group were also analyzed for response at week three (rapid responders) and week six (sustained response). At week three, 82% of the CDED group and 85% of the EEN group showed a response (PCDAI decrease > 12.5) or remission (PCDAI < 10). Remission at week three significantly increased the odds of remission at week six (OR 6.37, 95% CI: 1.6–25, *p* < 0.05), and week 12 (OR 4.5, 95% CI: 1.2–16.5, *p* < 0.05) [59,69].

Two further CDED studies were published in 2021. Real-world data was collected by Niseteo et al. to compare CDED to EEN [100]. The majority of patients received one to two weeks of EEN prior to starting CDED. There was comparable efficacy between CDED and EEN for induction of remission (66% EEN versus 75% CDED groups); however, patients who received CDED showed significantly higher weight gain. Yanai et al. performed the first adult CDED + PEN versus CDED study and showed that CDED (even without additional PEN) is a viable treatment option in mild-to-moderate (biologic naïve) CD [101]. Patients on CDED showed significant improvement in calprotectin and CRP, with sustained remission in 80% at 24 weeks and endoscopic remission in 35%.

#### 2.3.2. Effects of CDED on the Intestinal Microbiome

Clinical response in CDED was associated with changes in microbial composition and function, based on 16 s ribosomal ribonucleic acid (rRNA) and metagenomic sequencing analyses. In the Levine et al. 2019 RCT, remission in the CDED + PEN and EEN groups was associated with changes in microbial diversity, a decrease in Proteobacteria and an increase in Firmicutes [69]. However, changes at the genus level for Firmicutes phyla were not consistent across diets. Achieving remission at week six with CDED + PEN was associated with a significant increase in Firmicutes, particularly *Clostridiales*, driven by *Roseburia, Oscillibacter, Anaerotruncus* and *Ruminococcus.* Remission also led to a significant decrease in Proteobacteria, particularly Gammaproteobacteria. After week six (as patients entered Phase 2 of CDED + PEN), changes continued to be seen in the microbiome. A further decrease occurred in Proteobacteria up to week 12. Patients who did not achieve remission with CDED + PEN at week six showed fewer microbial changes; however, this was not statistically significant given the small number of children who failed to achieve remission [69,102].

When comparing children achieving remission, both CDED + PEN and EEN diets showed a decrease in Proteobacteria and an increase in Firmicutes. Although Proteobacteria abundance decreased, relative abundance was still high compared to healthy controls [76], particularly in *Escherichia coli* [102]. The increase in Firmicutes, although significant, was not consistent across genera between the two diets. Children receiving EEN showed increases in Firmicutes driven by *Clostridiales, Erysipelotrichaceae* and *Veillonellaceae* [102]. As both diets show a decline in Proteobacteria, this suggests that Proteobacteria might be important taxa in dietary interventions that affect intestinal inflammation in CD. The increase in the relative abundance of Firmicutes may therefore be due to secondary niche expansion following the decrease in Proteobacteria. This is supported by the observation that Firmicute expansion occurs with EEN, despite EEN not containing fibre, which is thought to be an important substrate that ordinarily supports Firmicute expansion [103].

#### 2.3.3. Summary

Emerging data suggest that CDED may be superior to EEN for the induction of clinical remission, based on improved tolerability profiles. Additional studies are needed to further describe the role of CDED as maintenance therapy and an adjunctive treatment alongside PEN and medications.

## 3. Diets without Proven Clinical Efficacy for IBD

### 3.1. Specific Carbohydrate Diet

The Specific Carbohydrate Diet (SCD) was developed based on research demonstrating the effect of complex carbohydrates consumption on intestinal microbiome dysbiosis and the subsequent influence on the intestinal mucosa and immune function [68]. Undigested disaccharides and polysaccharides promote the growth of proinflammatory species such as *Enterobacteriaceae*, as undigested carbohydrates remain within the colon to serve as a nutritional substrate. Conversely, the limited bioavailability of free carbohydrates inhibits the growth of butyrate-producing bacteria that elicit anti-inflammatory effects [104]. A diet rich in carbohydrates and fats contributes to the pathogenesis of IBD by nurturing proinflammatory taxa that foster mucosal thinning and permeability [105]. The SCD limits the intake of carbohydrates (grains, sugars (except honey)), dairy products (except fermented yogurt and hard cheeses) and processed foods while permitting the intake of monosaccharides (glucose, fructose, and galactose) [68]. Studies have shown that IBD patients who follow the SCD have increased diversity in their intestinal microbiota and normalization of intestinal inflammatory markers [106]. It is hypothesized that the SCD alters the fecal microbiome to favour less inflammatory taxa and ultimately reduces intestinal inflammation [107].

#### 3.1.1. Clinical Efficacy of SCD

There is a lack of high-quality studies assessing the effect of SCD. Suskind et al. reported results of an exploratory multiomics pilot study comparing the SCD, modified Specific Carbohydrate Diet (mSCD) and Whole Food diet (WF) in 18 paediatric patients with CD, of which only 10 completed the study [68]. All participants spent two weeks on the SCD prior to being randomized into one of three dietary treatment groups: SCD, mSCD, or WF. The mSCD allowed rice and oats while the WF eliminated wheat, corn, sugar, milk, and food additives. All meals were prepared by a study chef, and all patients were counselled by a dietician who specialized in SCD throughout the study. Clinical remission was seen more often in children completing the SCD after 12-weeks, but not with mSCD or WF diet. After 12 weeks, SCD was associated with decreases in PCDAI (21.9 ± 5.5 to 1.9 ± 3.8), whereas most children participating already had normal CRP at baseline (1.3 ± 0.7 mg/dL to 0.9 ± 0.5 mg/dL) and normal or mildly elevated erythrocyte sedimentation rate (ESR) (15.6 ± 13.7 mm/h to 13 ± 14.2 mm/h).

Patients in the mSCD group showed the greatest decrease in FC compared with the other groups (697 ± 520 mg/kg to 157 ± 156 mg/kg). Although children who followed the WF diet also showed a decrease in inflammatory markers, it was associated with weight loss, lethargy, hunger, and a higher rate of allergies. Participants from all three dietary interventions showed some improvement; however, the statistical significance of the results is limited due to the small sample size (*n* = 16), with even fewer patients completing the study (*n* = 10). Therefore, further research is required to determine the clinical response of SCD to Crohn’s disease. [68].

When compared to the Mediterranean Diet (MD) (see: Mediterranean Diet), Lewis et al. concluded that SCD is not a superior therapeutic option in the dietary treatment of IBD [47]. This 2021 RCT evaluated symptomatic remission, clinical remission and inflammatory biomarkers in adult patients with CD experiencing mild to moderate symptoms (*n* = 197). Participants were randomized to the SCD group (*n* = 101) or MD group (*n* = 96) and followed over 12 weeks. The primary outcome was symptomatic remission, and the secondary outcomes were FC response and CRP response. All participants were provided with prepared meals and snacks until week 6 of their assigned diet, after which they were asked to continue their diet independently. At week 12, symptomatic remission was achieved in 42.4% of the SCD group and 40.2% of the MD group (*p* = 0.87), while clinical remission was seen in 40.4% of the SCD group and 46.7% of the MD group (*p* = 0.28). Further, 26.1% and 7.7% achieved an FC response in the SCD and MD groups, respectively (*p* = 0.20). A CRP response was seen in only 10.8% of the SCD group and 7.1% of the MD group (*p* = 0.55).

There was no significant difference in the primary outcome between both groups, and combined symptomatic remission with the concurrent resolution of inflammation was rare. Although both diets were well tolerated, the SCD provides no additional benefit than the MD, and its restrictive nature may limit long-term adherence to the diet [47].

#### 3.1.2. Effects of SCD on the Intestinal Microbiome

Microbiome changes were variable among participants from the 2020 study by Suskind et al. [68]. Some studies have identified increased microbiome diversity in CD patients on the SCD, while others have used murine models to show that carbohydrate monotony provides a selective pressure favouring certain intestinal bacterial species while decreasing the total diversity [106,108]. Microbial composition in all three groups (SCD, mSCD, WF) showed a trend towards increased microbial diversity (Inverse Simpson Index), from 4.67–14.09 at enrollment to 10.93–17.76 at the end of the two-week study period [68]. Conversely, two patients had a modest decrease in microbial diversity (13% and 19%), and two patients had low diversity prior to initiating the study.

Across all cohorts, *Blautia*, *Lachnospiraceae, Faecalibacterium, Roseburia, Anaerobutyricum* and *Eubacterium* were enriched; *Escherichia coli* and *Faecalibacterium prausnitzii* had decreased abundance. Although less pronounced, stool metagenomics analyses showed increased metabolic activity in the mSCD cohort associated with starch and sucrose metabolism and increases in amino acid metabolism. The WF diet cohort showed increased amino acid metabolism and variable changes in glutamate synthase, methionine synthase, and 2-dehydro-3-deoxy-D-pentonate aldolase enzyme activity. These alterations in microbiome activity are indicative of adaptions to dietary changes and impact the immune system by abating intestinal inflammation. However, the mechanism by which these changes occur remains unclear [68].

#### 3.1.3. Summary

There is a lack of high-quality studies supporting SCD. Existing data are limited by small sample sizes, and in the largest study (DINE-CD), no significant differences were found between the SCD and MD [47]. Current evidence does not suggest a role for the SCD in the induction or maintenance of remission for CD or UC.

### 3.2. Crohn’s Disease Treatment with Eating Diet (CD-TREAT)

The Crohn’s Disease TReatment with EATing diet (CD-TREAT) was developed by Svolos et al. to target an unmet need for a real food diet that was similar in nutritional composition to exclusive enteral nutrition (EEN) but more acceptable to patients [46]. The diet was based on the composition of Modulen^®^ formula (Nestle^®^, Vevey, Switzerland), but instead used “real” food that was prescribed and individualized according to patients’ food preferences. It omits gluten, lactose, and alcohol and matches other macronutrients, vitamins, minerals, and fibre [75,77,79].

The CD-TREAT study involved healthy volunteers, an animal model, and an open-label pilot trial [46]. Healthy adult control subjects were started on EEN (Modulen^®^) or CD-TREAT for seven days, followed by a 14-day washout period, then switched to the alternate diet for another seven days. In the open-label arm, the authors assessed the efficacy of CD-TREAT at inducing remission in a pilot trial of five children with active CD over eight weeks. Four patients (80%) showed clinical improvement (decrease in weighted-PCDAI [wPCDAI], score of ≥17.5) and three patients (60%) achieved clinical remission (wPCDAI < 12.5) with a concurrent decrease in fecal calprotectin (FC) (mean decrease 918 ± 555 mg/kg; *p* = 0.002) [46].

Effects on the intestinal microbiome in healthy adults were similar in the EEN and CD-TREAT groups in terms of microbial composition, mean total sulphide content, pH, and SCFA levels. Effects on the microbiome were not assessed in the paediatric treatment group [46]. The authors concluded that the CD-TREAT diet could achieve similar effects on the microbiome as EEN in both healthy adults and experimental rat models. The report of the larger clinical trial is eagerly awaited and will hopefully add another option to the dietary treatments of CD [106,108,109].

#### Summary

The CD-TREAT diet is still in the very early stages of assessment. Therefore, no conclusions about its efficacy for the induction or maintenance of remission in CD or UC should be made yet.

### 3.3. Mediterranean Diet (MD)

The Mediterranean diet (MD) consists of a high intake of fruit, vegetables, legumes, nuts, whole grains, and olive oil. It includes moderate fish consumption and a low intake of saturated fat, meat, and dairy products [110,111]. MD has been recognized for its potential benefits in IBD [46,110,112,113,114]. Limited data in children have suggested an association with lower inflammation measured by FC [114]. A study of healthy Italian adults provided evidence for dietary intake and its impact on the microbiome and associated metabolome. In subjects who were broadly adherent to the MD, the study demonstrated an increase in fecal short-chain fatty acid (SCFA) levels and a higher proportion of fibre-degrading bacteria belonging to Firmicutes and Bacteroidetes phyla. In contrast, low adherence to the MD corresponded with higher urinary trimethylamine oxide (TMAO) levels [115].

#### 3.3.1. Clinical Efficacy and Effects on the Microbiome

The DINE-CD trial (see: SCD) compared the efficacy of the SCD to the MD in adults with CD. This study was designed to test the hypothesis that the SCD is superior to the MD and hence was powered as a superiority trial. Patients with mild to moderate CD, defined as a short Crohn’s disease Activity Index (sCDAI) score >175 and <400, were randomized to receive either the SCD (*n* = 101) or the MD (*n* = 93). Patients received six weeks of delivered meals and snacks, followed by six weeks of independent meal preparation based on instructions from the study team with recommended ingredients. SCD was not found to be superior to the MD. DINE-CD showed no significant difference in clinical remission rates or change in FC. However, only a minority of patients had elevated FC at baseline, suggesting that patients who entered the trial had very mild disease. Given the comparative effectiveness design of this trial and the lack of a control arm, this study does not help assess the overall efficacy of either diet or show any benefit or inferiority compared to an individual’s usual diet [47].

The association of low adherence to the MD and TMAO may be significant. Decreased TMAO levels are seen in IBD compared to a non-IBD population [116]. TMAO is produced by gastrointestinal anaerobes through the digestion of dietary phosphatidylcholine and carnitine. This compound may serve as a biomarker for IBD [116]. TMAO has been found to increase following fecal microbiota transplant (FMT) in patients with Crohn’s disease prior to their second dose of FMT [117]. In a study of eight adults with CD on six weeks of a “Mediterranean-inspired diet,” significant changes in gene expression were observed. While patients self-reported adherence, results were compelling. While no single gene stood out, metagenomics analysis showed a change in expression of >3500 genes whose cumulative effects have been associated with “normalizing” intestinal microbiota to a composition more similar to healthy controls than patients with IBD, with an increased abundance of Bacteroidetes and a decrease in Proteobacteria and *Bacillaceae* [83].

With the exception of CDED, high-quality data to guide real food dietary recommendations are lacking [118]. If the CDED is not being considered to treat an individual with CD, then the MD, given its relative ease and its additional reported health benefits, may be a sensible dietary recommendation for patients seeking general advice on nutrition choices in IBD [112,114,115].

#### 3.3.2. Summary

There are insufficient data to support the role of the MD for the induction or maintenance of remission in CD or UC. The primary study assessing MD was a head-to-head study comparing MD to SCD, which also does not demonstrate efficacy. Without further prospective data or randomized controlled trials, the MD cannot be recommended in the management of IBD at this time.

### 3.4. Low FODMAP Diet (LFD)

A Low FODMAP Diet (LFD) involves the restriction of fermentable oligosaccharides, disaccharides, monosaccharides, and polyols (FODMAP), which are poorly digested by the proximal gastrointestinal tract and readily fermented by colonic bacteria. This diet is thought to provide symptomatic relief by reducing colonic gas and luminal distension [71]. LFD is described most commonly as a treatment option for irritable bowel syndrome (IBS) by reducing abdominal pain, bloating, and diarrhea [72]. Conversely, it is hypothesized that LFD can promote intestinal inflammation by reducing the prebiotic effect of FODMAPS on the colonic microbiome and consequently impact the microbiome’s immunomodulatory effects on the mucosal barrier [72].

#### 3.4.1. Clinical Efficacy of LFD

In a recent randomized controlled trial at two large gastroenterology clinics in the United Kingdom, the LFD resulted in significant symptomatic improvement of IBS in adult patients with quiescent IBD [71]. Overall, 52% of participants in the LFD cohort experienced relief of symptoms after four weeks, compared with only 16% in the sham (placebo) diet control. Furthermore, the effects of GI symptoms on health-related quality of life, as determined by the Bowel II domain score on the IBD questionnaire, was significantly greater with the LFD.

There was no change seen in inflammatory markers. Specifically, there was no statistically significant difference in FC or CRP at the end of the trial with either the LFD or control. Interestingly, the IBD-control score demonstrated that participants with ulcerative colitis experienced greater patient-perceived control of their disease with the LFD (88.3, SEM 4.3) compared with the sham diet (74.3, SEM 4.5). Short-chain fatty acids were also lower in the UC group on LFD. These findings were not seen in the CD population [71].

A second RCT assessed the LFD (*n* = 26) versus a standard diet (*n* = 29) on IBD patients over a six-week study period [72]. There was a significant difference in median FC levels, which decreased by 34.4% in the LFD group and 4.4% in the standard diet group, but there were no differences in CRP levels. Although the LFD may reduce the symptomatic burden of IBS in IBD, more extensive series and longer study periods are needed to understand its effect, if any, on inflammatory pathways.

#### 3.4.2. Effects of LFD on the Intestinal Microbiome

Functional metagenomics highlighted 34 Kyoto Encyclopedia of Genes and Genomes (KEGG) Orthology (KO) groups that varied between LFD and sham diet controls. Out of 616 identified species in the intestinal microbiome, 29 were significantly affected by the LFD (*p* ≤ 0.05). Following targeted microbiome analysis, the total population of *Bifidobacteria* remained unaltered; however, the composition varied within the LFD group. Strains such as *Bifidobacterium longum* and *Bifidobacterium adolescentis* were significantly lower, while *Bifidobacterium dentium* was higher. Additionally, the total population of *Faecalibacterium prausnitzii* decreased without any change in specific strains [71].

#### 3.4.3. Summary

Existing data do not support the efficacy of the LFD for the induction or maintenance of remission in CD or UC; however, LFD may have a role in symptomatic improvement. In two RCTs that have been conducted, there was no change in inflammatory markers, but the symptomatic improvement was shown. Neither study described the impacts on nutritional micronutrient status in study participants. Given the restrictive nature of the LFD, nutritional sufficiency would be an important outcome to assess in future studies.

### 3.5. Miscellaneous Diets

Multiple studies have described other miscellaneous dietary interventions for the management of IBD. A Cochrane Systematic Review evaluated the strength of evidence across these studies and concluded that the majority of them were inadequately powered, and therefore no firm conclusions could be drawn regarding their benefits or harms [118].

Albenberg et al. studied the role of diets low in red and processed meat [119]. Based on self-reported consumption over 49 weeks, groups were assigned to high meat intake (minimum of two servings per week of red or processed meat, *n* = 118) or low meat intake (<2 servings per month of red or processed meat, *n* = 96). Disease relapse occurred in 62% of participants in the high meat intake group and 42% of participants in the low meat intake group. There was no significant difference in time to relapse (*p* = 0.61), or severity of relapse (*p* = 0.50).

Two studies assessed the role of a cow’s milk protein (CMP) elimination diet on IBD. A 2019 study by Strisciuglio et al. randomized 29 consecutive paediatric patients (mean age: 11.2 years) with newly diagnosed UC to a CMP-free diet or a liberalized diet [25]. At one year of follow-up, 53.8% of patients treated with the CMP elimination diet and 53.3% receiving a liberalized diet relapsed (*p* = 1.0). A 1965 study by Wright et al. had similar findings [24]. Forty-nine adult UC patients were randomized to a CMP-free diet versus liberalized diet. A total of 61.5% of patients in the CMP-free group versus 78.3% of patients in the liberalized diet group experienced a relapse at 12 months, suggesting no significant effect of CMP avoidance in preventing disease relapse.

A recent 2021 prospective cohort study by Narula et al. offered additional evidence for the impact of WD on the development of IBD. This international, multicentre observational cohort assessed associations between ultraprocessed food intake and IBD in 116 087 adults ages 35–70 years by reporting baseline food frequency questionnaires obtained between 2003 and 2016 [120]. A higher intake of ultra-processed foods, defined as soft drinks, refined sweetened foods, salty snacks, and processed meats, was associated with a higher risk of the development of IBD (HR 1.82, 95% CI: 1.22 to 2.72 for ≥5 servings/day; and HR 1.6, 95% CI: 1.18 to 2.37 for 1–4 servings/day; compared with <1 serving/day; *p* = 0.006). Intake of white meat, red meat, dairy, starch, fruit, vegetables, and legumes was not found to be associated with the development of IBD. Results were consistent between CD and UC development. These results may explain the global trend of increasing rates of IBD among industrialized nations, where there is significantly higher availability of ultra-processed foods containing additives and preservatives [121,122]. In turn, specific dietary patterns have been associated with more proinflammatory microbiome features and the occurrence of flares.

A recent 2021 pilot study by Sarbagili-Shabat et al. assessed the feasibility and efficacy of a novel UC exclusion diet (UCED) aimed at minimizing dietary components described to adversely impact goblet cells, mucosal permeability, and microbial composition [123]. This study assessed the efficacy of UCED for the induction of clinical remission in paediatric UC. Twenty-three children (mean age 15.3 ± 2.9 years) were administered the UCED over six weeks, with median paediatric ulcerative colitis activity index (PUCAI) decreasing from median 35 to 12.5 (*p* = 0.001), clinical remission was achieved in 37.5% (9/24), and fecal calprotectin decreased from 818 (630–1880 µg/g) at baseline to 592 (141–1555 µg/g) at week 6 (*p* > 0.05). While preliminary, the UCED shows efficacy for the induction of remission in mild to moderate paediatric UC. This data further suggests the emerging role of diet as induction therapy for UC.

## 4. Further Considerations for Dietary Therapy

There are several important considerations for patients seeking nutritional therapy. First, the safety of the dietary intervention is paramount: high fibre dietary intake may precipitate (sub)obstructive symptoms in stricturing CD. If these (sub)obstructive symptoms such as vomiting (especially when bilious) and cramping abdominal pain occur, then a surgical consult is warranted and if continued therapeutic feeding is deemed appropriate, EEN may be considered the preferred therapy.

Second, the feasibility of the dietary intervention is paramount. EEN has the greatest published efficacy for treating CD, but dietary monotony, formula costs, and the social challenges of nasogastric tube feeding and food avoidance make it a challenge for many patients. When considering treatment risks and benefits, social, psychological, and financial risks are equally important to consider.

Third, nutritional sufficiency is an important consideration, particularly in children and adolescents, which can be challenging when many foods are excluded. IBD and its effects on intestinal absorption can result in significant baseline and ongoing nutrient deficiency. Highly circumscribed diets that require multiple food avoidances need to be followed under the close supervision of a multidisciplinary IBD team, including a registered dietitian. Patients should be checked for caloric, macronutrient, and micronutrient sufficiency regularly, and deficiencies promptly corrected when identified.

Finally, there is a need for higher quality data in the nutrition sciences [124]. Variability in dietary adherence, error-prone self-reporting of food frequency and food intake, and heterogeneous baseline characteristics of participants significantly affect the interpretation of studies [118]. Given these limitations, future studies should address the role of diet as add-on therapy to medical therapy, including for moderate-to-severe disease, as recently reported in both CD and UC [61,63]. Individual IBD patients commonly report highly variable food sensitivities with associated short-term symptoms such as bloating, cramping, nausea, constipation or diarrhea, which should be distinguished from dietary pattern associated changes on intestinal inflammation and microbiome composition.

## 5. Conclusions

Food and nutrition have become an important focus in people’s lives. This is particularly true for patients with gastrointestinal diseases. As the incidence of IBD continues to rise worldwide, patients will continue to seek opportunities for autonomy and self-management of their disease. Dietary interventions offer an attractive choice, giving patients a degree of self-efficacy amidst their many other prescribed treatments, in addition to postponing or limiting medical treatment side effects and long-term health implications. It is important to recognize this and provide strong, evidence-based guidance for patients. This review describes several valuable options for patients. Moreover, the evolving evidence for dietary interventions in the treatment of IBD underscores the critical relationship between diet, the microbiome, and immune regulation. It remains unclear whether the microbial shifts observed with these dietary interventions are responsible for the effectiveness of dietary therapies or are a consequence of reduced intestinal inflammation favouring different luminal taxa. Until future research can untangle these associations, IBD patients should continue to view their nutritional intake as an important contributor to their overall disease management.

## Figures and Tables

**Table 2:** Reported taxonomic changes by nutritional therapy.

Type of Diet	Taxa Increased	Taxa Decreased	Clinical Efficacy
EEN [52,65,77,79,81,82]	Firmicutes (p): Clostridiales (c), Erysipelotrichaceae (c), *Veillonellaceae* (f)	Proteobacteria (p) Actinobacteria (p): *Bifidobacterium* (g), *Ruminococcus* (g), *Faecalibacterium* (g), *Faecalibacterium* *prausnitzii* (s) Bacteroidetes (p): *Bacteroides* (g), *Prevotella* (g)	80–85% remission rate Equivalent to CS for clinical remission Small studies show EEN superior to CS for mucosal healing
PEN	No data available	No data available	No evidence of efficacy for induction of remission The systematic review demonstrates efficacy for maintenance of remission
CDED [69,70]	Firmicutes (p): Clostridiales (c), *Roseburia* (g), *Oscillibacter* (g), *Anaerotruncus* (g), *Ruminococcus* (g)	Actinobacteria (p) Proteobacteria (p): Gammaproteobacteria (c)	70–75% remission rate in children at week six 76% remission rate in children at week 12
SCD [68]	Fusobacteria (p): *Fusobacterium ulcerans* (s) Firmicutes (p): Clostridiales (c), *Eubacterium* (g), *Blautia* (s), *Lachnospiraceae* (f), *Roseburia* (g), *Anaerobutyricum* (g), *Faecalibacterium* (g)	Proteobacteria (p): *Escherichia coli* (s) Actinobacteria (p): *Faecalibacterium* *prausnitzii* (s)	All patients showed clinical improvement and FC improvement SCD showed the greatest clinical improvement mSCD showed the greatest FC change Cannot conclude significance with sample size
CD-TREAT	No data available	No data available	80% (four out of five) clinical improvement 60% (three out of five) clinical remission, improved FC
MD [83]	Firmicutes (p) Bacteroidetes (p)	Proteobacteria (p) Firmicutes (p): *Bacillaceae* (f)	No significant difference in clinical symptoms, FC values between MD and SCD
LFD [71,72]	Actinobacteria (p): *Bifidobacterium dentium* (s)	Actinobacteria (p): *Faecalibacterium* *prausnitzii* (s), *Bifidobacterium longum* (s), *Bifidobacterium* *adolescentis* (s)	52% decrease in symptoms 34% decrease in FC

Crohn’s disease exclusion diet (CDEC); Crohn’s disease treatment-with-eating diet (CD-TREAT); corticosteroids (CS); exclusive enteral nutrition (EEN); fecal calprotectin (FC); low fermentable oligo-, di-, monosaccharides and polyol (FODMAP) diet (LFD); Mediterranean diet (MD); partial enteral nutrition (PEN); specific carbohydrate diet (SCD); phylum (p); class (c); family (f); genus (g); species (s).

**Table 3:** Clinical trials actively recruiting for nutritional therapies for patients with CD.

Name	Intervention	Country
The Intensive Post Exclusive Enteral Nutrition Study	CD-Treat	UK
Diet for Induction and Maintenance of Remission and Rebiosis in Crohn’s Disease	EEN, mEEN, PEN, CDED	Canada, Ireland, Israel, Spain, Netherlands
“Tasty & Healthy” Dietary Approach for Crohn’s Disease	Whole food diet	Israel
The Challenge Study: A Dietary Personalization Protocol for Patients with Crohn’s Disease and Deep Remission	CDED + milk fat and gluten	Israel
Exclusive Enteral Nutrition versus Infliximab in Chinese CD Patients	EEN	China
Biologics and Partial Enteral Nutrition Study	PEN	UK, Scotland
Adherence to Exclusive Enteral Nutrition in Patients with Crohn’s Disease	EEN	China
Based on the Special Disease Management of Crohn’s Disease Diet Studies	CD-C food	China
Diet in Paediatric Crohn’s Disease Treated with Biologics	CDED	Argentina

Inflammatory bowel disease (IBD); Crohn’s disease exclusion diet (CDED); Crohn’s disease treatment-with-eating diet (CD-TREAT); exclusive enteral nutrition (EEN); modified exclusive enteral nutrition (mEEN); partial enteral nutrition (PEN); Crohn’s disease Chinese food (CD-C food).
